# Deep Learning Improved Clinical Target Volume Contouring Quality and Efficiency for Postoperative Radiation Therapy in Non-small Cell Lung Cancer

**DOI:** 10.3389/fonc.2019.01192

**Published:** 2019-11-13

**Authors:** Nan Bi, Jingbo Wang, Tao Zhang, Xinyuan Chen, Wenlong Xia, Junjie Miao, Kunpeng Xu, Linfang Wu, Quanrong Fan, Luhua Wang, Yexiong Li, Zongmei Zhou, Jianrong Dai

**Affiliations:** ^1^Department of Radiation Oncology, National Cancer Center/National Clinical Research Center for Cancer/Cancer Hospital, Chinese Academy of Medical Sciences and Peking Union Medical College, Beijing, China; ^2^National Cancer Center/National Clinical Research Center for Cancer/Cancer Hospital and Shenzhen Hospital, Chinese Academy of Medical Sciences and Peking Union Medical College, Shenzhen, China

**Keywords:** non-small cell lung cancer, postoperative radiotherapy, clinical target volume, deep learning, automatic contour

## Abstract

**Purpose:** To investigate whether a deep learning-assisted contour (DLAC) could provide greater accuracy, inter-observer consistency, and efficiency compared with a manual contour (MC) of the clinical target volume (CTV) for non-small cell lung cancer (NSCLC) receiving postoperative radiotherapy (PORT).

**Materials and Methods:** A deep dilated residual network was used to achieve the effective automatic contour of the CTV. Eleven junior physicians contoured CTVs on 19 patients by using both MC and DLAC methods independently. Compared with the ground truth, the accuracy of the contour was evaluated by using the Dice coefficient and mean distance to agreement (MDTA). The coefficient of variation (CV) and standard distance deviation (SDD) were rendered to measure the inter-observer variability or consistency. The time consumed for each of the two contouring methods was also compared.

**Results:** A total of 418 CTV sets were generated. DLAC improved contour accuracy when compared with MC and was associated with a larger Dice coefficient (mean ± SD: 0.75 ± 0.06 vs. 0.72 ± 0.07, *p* < 0.001) and smaller MDTA (mean ± SD: 2.97 ± 0.91 mm vs. 3.07 ± 0.98 mm, *p* < 0.001). The DLAC was also associated with decreased inter-observer variability, with a smaller CV (mean ± SD: 0.129 ± 0.040 vs. 0.183 ± 0.043, *p* < 0.001) and SDD (mean ± SD: 0.47 ± 0.22 mm vs. 0.72 ± 0.41 mm, *p* < 0.001). In addition, a value of 35% of time saving was provided by the DLAC (median: 14.81 min vs. 9.59 min, *p* < 0.001).

**Conclusions:** Compared with MC, the DLAC is a promising strategy to obtain superior accuracy, consistency, and efficiency for the PORT-CTV in NSCLC.

## Introduction

Accurate delineation of the clinical target volume (CTV) is one of the most crucial aspects of treatment planning in radiotherapy. The quality of this process depends on the expertise of the individual observer and is associated with quality outcomes in lung cancer care (1–4). However, significant inter-observer variation, even among experts in clinical trials with specific protocols, has been reported (4–6). In less-developed countries that lack a sufficient number of facilities and training opportunities for radiation oncologists, there is even greater disparity in clinical expertise among physicians from different areas (7).

Computer-aided tools can potentially improve contour accuracy and reduce variation. Recently, deep learning methods have been increasingly involved in the field of radiotherapy (8–19). Our group has also previously established a series of auto-segmentation models in breast cancer and rectal cancer that use big data and deep learning (10, 11). As a state of the art technique, deep learning has manifested superior performances by enabling self-taught discovery of informative representations and using hierarchical layers of learned abstraction to accomplish high-level tasks efficiently (10). Another important advantage of deep learning methods is that they are suitable for various patient body sizes and shapes. Even if input images show huge differences in body size or shape, deep learning can increase knowledge from a sufficient number of examples and produce excellent segmentation results (10, 11).

With regard to lung cancer, deep learning-related published data have predominantly focused on organs at risk (OAR) or gross tumor contouring (14–18, 20, 21) and have consistently outperformed the existing solutions in most settings but still require final manual modification before the eventual clinical implementation (14, 20, 21). However, few studies have explored the role of this cutting-edge technique for CTV contouring in patients who received postoperative radiation therapy (PORT).

In this study, we established a robust deep learning algorithm to segment the CTV for non-small cell lung cancer (NSCLC) receiving PORT and determined if the assistance of deep learning could provide greater accuracy, inter-observer consistency, and contouring efficiency than those of manual delineation.

## Materials and Methods

### Patient Selection

We selected 250 pN2-NSCLC patients who received PORT in our department between 2012 and 2016 to build the deep learning model, with 200 patients randomly assigned to a training set and 50 to the validation set. We then selected 19 patients treated between 2016 and 2018 in our department as the test cohort to generate the automatic delineation of the CTV. All patients were simulated in the supine position with both arms raised above the head. The thickness of all scanned slices was 5 mm, and all computed tomography (CT) images were transferred using the Digital Imaging and Communications in Medicine (DICOM) format. The CT data were acquired on a Somatom Definition AS 40 (Siemens Healthcare, Forchheim, Germany) or on Brilliance CT Big Bore (Philips Healthcare, Best, the Netherlands) systems.

### Deep Learning for Segmentation

We introduced a robust deep learning algorithm based on ResNet-101 to segment the CTV for PORT patients, which has been proven to provide high performance in CTV delineation (11). An end-to-end segmentation framework was able to predict pixel-wise class labels in the CT images. The dilated module with different atrous rates was able to extract multi-scale features from the CT, which led to a more robust model. The training, validation, and testing were implemented in Caffe by using the GeForce® GTX 1080 Ti graphics card (22). The inputs to the deep learning model were the two-dimensional CT images, and the outputs were the corresponding labels of the CTV. The training set (comprising CT images and manual segmentation labels) was used to tune the parameters of the network. Data augmentation methods, such as random cropping, rotation, scaling, and contrast adjustment, were adopted to enlarge the training set. The following training strategy of the network was used: batch size of 1, momentum of 0.9, weight decay of 0.0005, learning rate policy of poly, initial learning rate of 0.001, power of 0.9, and training iterations of 80 K. The model with the highest performance on the validation set was selected as the final model.

### Ground Truth Contours

Three senior radiation oncologists (ZZ, NB, and JW with work experience > 10 years) also contoured the CTV in accordance with the protocol of a randomized phase III trial of the PORT study primarily investigated by our institute (NCT00880971) while blinded to the others' work on the same set of PORT images. The majority voting was used to generate the ground truth (GT).

### Contour Methods

Eleven junior radiation oncologists (working experience ≤ 5 years) from 11 institutions with various volumes of radiation oncology departments (detailed information of the departments' volumes is presented in Supplementary Table 1) were selected to independently contour the CTV on the same set of PORT images. Each radiation oncologist created two contour sets of the CTV in this study: (1) a manual contour (MC) and (2) a deep learning-assisted contour (DLAC). All MC tasks were performed in the treatment planning system (TPS) Pinnacle 9.10 (Philips Radiation Oncology Systems, Fitchburg, WI). In terms of the DLAC, the physicians first ran the auto-contouring script, which could enable the automatic delineation of the PORT-CTV on the CT images using the previously trained model, and then wrote the contours into the DICOM RT structures file of the TPS. Afterwards, the physicians manually adjusted these automatic contours in the TPS at their discretion to generate the DLAC. For each contouring task, the starting and ending time was recorded by using an in-house script. The interval time between them was defined as the contouring time, with an accuracy of 1 s.

### Accuracy Assessment

The accuracy of the junior radiation oncologists' delineation was evaluated by using the Dice coefficient and mean distance to agreement (MDTA) with the GT as a reference (14, 23–25). The Dice coefficient is defined as:

$$\begin{array}{l} \text{Dice coefficient}=2\left(V_{\text{J}}\cap V_{\text{GT}}\right)/\left(V_{\text{J}}+V_{\text{GT}}\right) \end{array}$$

where V_J_ represents the CTV contoured by each junior physician, and V_GT_ represents the ground truth CTV created by the three senior physicians. A value of 1 indicates a perfect concordance between two contours. MDTA is the mean distance between the surfaces of both volumes, with a value of 0 representing perfect agreement. Both Dice coefficient and MDTA were generated in MIM software (version: 6.9.2, Cleveland, OH).

### Inter-observer Consistency Assessment

The inter-observer consistency was assessed using both volume and spatial metrics. The coefficient of variation (CV) was defined as the standard deviation (SD) divided by the mean CTV volume of all observers for each patient contoured with each delineation method, where a larger CV indicates greater variability or lower consistency. For the spatial metrics, the standard distance deviation (SDD) was used to measure the dispersion of the centroid distribution of CTVs contoured by the 11 junior radiation oncologists, which represents the standard deviation of the distance of each point from the mean center (26):

$$\begin{array}{l} Xc=\frac{\sum_{i=1}^{n}Xi}{n}\;\\ Yc=\frac{\sum_{i=1}^{n}Yi}{n}\;\\ Zc=\frac{\;\sum_{i=1}^{n}Zi}{n}\;\\ SDD=\sqrt{\frac{\sum_{i=1}^{n}{\left(Xi-Xc\right)}^{2}+\sum_{i=1}^{n}{\left(Yi-Yc\right)}^{2}+\sum_{i=1}^{n}{\left(Zi-Zc\;\right)}^{2}}{N}} \end{array}$$

where *X*_*i*_*, Y*_*i*_*, and Z*_*i*_ represent the coordinates of the centroids of the CTVs created by the junior radiation oncologists and can be obtained from the MIM. *X*_*c*_*, Y*_*c*_, and *Z*_*c*_ represent the coordinates of the mean center of a set of centroids of CTVs for each patient delineated by the 11 junior radiation oncologists using one specific contour method. The unit of SDD is cm and a larger SDD indicates a greater systematic shift across all of the radiation oncologists.

### Statistical Analysis

The continuous variables were presented as the mean ± SD or median (interquartile range), which depended on the normality of the data. Correspondingly, the *t*-test or Mann–Whitney *U*-test were used to compare the variables between two contouring methods. These analyses were all performed in SPSS version 19.0 (SPSS, Inc. IBM, Armonk, NY, USA). All tests were two sided, and *p* ≤ 0.05 was considered to be indicative of statistical significance.

## Results

### General Characteristics of Study Patients

General characteristics of the patients in the test cohort are demonstrated in Table 1. The median age was 52 and the dominant pathology was adenocarcinoma. Primary location of tumors included 42.1% in upper lobe, 10.5% in middle lobe, and 47.4% in lower lobe. All patients had N2 stage and 52.6% had T2 stage of disease. More than half of the patients carried ≥ 3 stations of lymph node involvement and the median number of metastatic lymph node was 6.

**Table 1:** General characteristics of study patients.

**Characteristics**		**Number (*n* = 19)**
Age	Median (Range)	52 (35, 66)
Gender	Male	10 (52.6%)
	Female	9 (47.4%)
Pathology	Adenocarcinoma	18 (94.7%)
	Squamous cell carcinoma	1 (5.3%)
Primary Lobe	Upper	8 (42.1%)
	Middle	2 (10.5%)
	Lower	9 (47.4%)
T Stage	T1	6 (31.6%)
	T2	10 (52.6%)
	T3	2 (10.5%)
	T4	1 (5.3%)
Number of involved nodal station	1	3 (15.8%)
	2	6 (31.6%)
	≥ 3	10 (52.6%)
Number of resected lymph node	Median (Range)	19 (10, 40)
Number of involved lymph node	Median (Range)	6 (1, 17)

### Descriptive Statistics of CTV Volume

**Figure 1:**
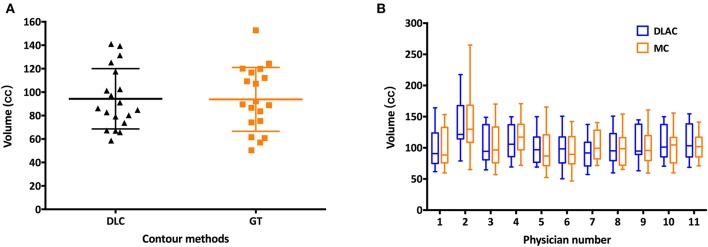
**(A)** Scatter plots of the volume measurements of the senior radiation oncologists' contours for each patient with a DLC (black triangle) and MC (yellow square) and **(B)** summary of the junior radiation oncologists' contours for each patient with a DLAC (blue boxes) and MC (yellow boxes), with whiskers from the 5–95th percentiles. DLC, deep learning contour; DLAC, deep learning-assisted contour; MC, manual contour.

**Figure 2:**
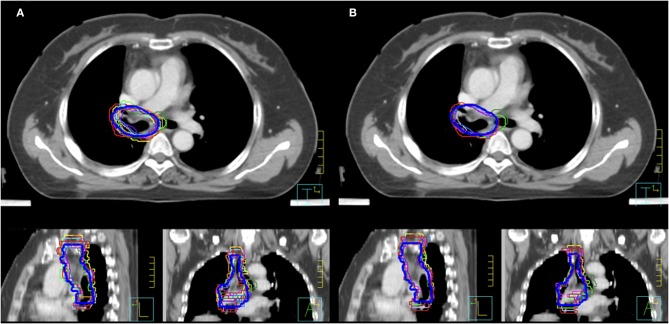
Clinical target volume delineated by the 11 junior radiation oncologists and GT contours (blue bold line) for a representative patient with an MC **(A)** and a DLAC **(B)**. DLAC, deep learning-assisted contour; MC, manual contour; GT, ground truth contour.

As shown in Figure 1A, the mean volume of the deep learning contour-based CTV (DLC) and GT-CTV was 94.30 cc (SD: 25.06 cc) and 93.78 cc (SD: 24.70 cc), respectively (*p* = 0.43). As for juniors' contour, a total of 418 unique CTV sets were generated, with 209 sets (19 × 11 = 209) for the MC and DLAC, respectively. The volume measures for each junior radiation oncologist with DLAC and MC are shown in Figure 1B and no volume difference was observed between two methods (*p* = 0.49). Figure 2 shows the CTV contours of the 11 junior radiation oncologists with the DLAC and MC and the GT contours for a representative patient.

### Accuracy Analysis

**Figure 3:**
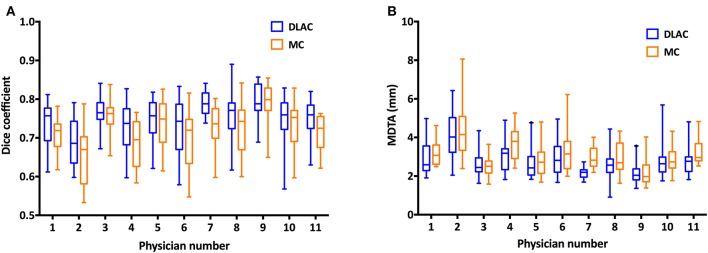
**(A)** Dice coefficient of the contours with a DLAC (blue boxes) and MC (yellow boxes) and **(B)** Mean distance to agreement (MDTA) of the contours with a DLAC (blue boxes) and MC (yellow boxes), compared with the GT contour for each junior radiation oncologist with whiskers from the 5–95th percentiles. DLAC, deep learning-assisted contour; MC, manual contour; GT, ground truth contour.

Using GT contours as the reference, the Dice coefficient of each junior radiation oncologist for the DLAC and MC is graphed in Figure 3A, which revealed a greater Dice coefficient for the DLAC than for the MC (mean ± SD: 0.75 ± 0.06 vs. 0.72 ± 0.07; *p* < 0.001). Similarly, the DLAC also presented a smaller MDTA per individual CTV set than that of the MC performed by the junior radiation oncologists (mean ± SD: 2.97 mm ± 0.91 mm vs. 3.07 mm ± 0.98 mm; *p* < 0.001), as shown in Figure 3B.

### Inter-observer Consistency Analysis

**Figure 4:**
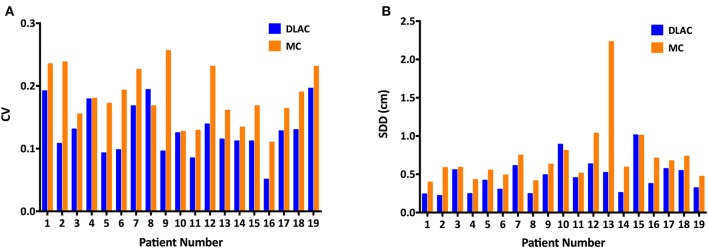
**(A)** Coefficient of variation (CV) of the clinical target volume and **(B)** Standard distance deviation (SDD) of centroids for each patient with the DLAC (blue bars) and MC method (yellow bars). DLAC, deep learning-assisted contour; MC, manual contour.

For volume metrics of inter-observer variability, the DLAC introduced a remarkably lower CV than that of the MC (mean ± SD: 0.129 ± 0.040 vs. 0.183 ± 0.043; *p* < 0.001; Figure 4A), which resulted in a 30% reduction of the CV. With regard to spatial metrics, the DLAC was associated with a significantly smaller SDD than that of the MC (mean ± SD: 0.47 ± 0.22 mm vs. 0.72 ± 0.41 mm; *p* < 0.001), which led to a 35% decrease in the SDD (Figure 4B).

### Contouring Time Analysis

**Figure 5:**
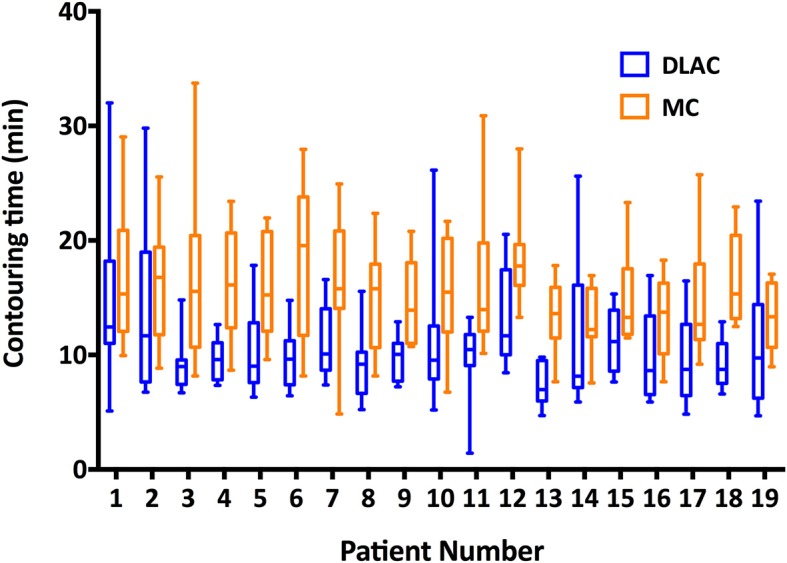
Contouring time of the DLAC (blue boxes) and MC (yellow boxes), with whiskers from the 5–95th percentile displayed for each individual patient. DLAC, deep learning-assisted contour; MC, manual contour.

Figure 5 shows the contouring time of the two methods for each patient. The median contouring time of the junior radiation oncologists when using the MC and DLAC for one patient were 14.81 min (interquartile range: 11.88, 18.64) and 9.59 min (interquartile range: 7.64, 11.91), respectively, which resulted in a 35% reduction (absolute: 5.22 min) in time consumption with assistance of the DLC (*p* < 0.001; Figure 5).

## Discussion

Accurate delineation of the CTV is one of the most crucial aspects of treatment planning in radiation therapy, whereas the quality of CTV delineation largely depends on the academic expertise of the individual physician. In our country, with a shortage of facilities and workforce, there is inevitably a great disparity in clinical expertise across physicians from different areas (7). The introduction of an advantageous automatic contouring tool has been strongly expected to improve contour accuracy as well as reduce contour discrepancy and time consumption. To the best of our knowledge, this is the first study to evaluate the performance of a DLAC of CTV for patients receiving PORT. As hypothesized, our results demonstrated superior accuracy and inter-observer consistency, and time saving resulted from the introduction of deep learning.

For pN2 NSCLC patients receiving a complete resection, the role of PORT is still open to debate. Notwithstanding the paucity of randomized data, a majority of evidence suggests that selected high-risk patients could benefit from PORT in terms of both local control and OS (27–33). For a target-generating study, the contours delineated by experienced expert physicians from high-volume institutions is generally considered GT as the reference (13, 14). Therefore, the increased consistency between the evaluated contours and the GT indicates an improvement in the contour accuracy. In the present study, the GT contours were generated based on the contemporary re-delineation by the three senior radiation oncologists as per the protocol of a randomized phase III trial of a PORT study primarily investigated by our institute, rather than by simply using the previous contours administered in clinical practice. This merit would be meaningful in avoiding the potential inter-observer variation among multiple senior physicians who created those contours for clinical practice. The MDTA and the Dice coefficient are widely accepted parameters for the evaluation of consistency between different segmentations (12, 14). The MDTA is a distance parameter, whereas the Dice coefficient is a volume overlap index, with a smaller MDTA and higher Dice coefficient indicating a greater contour accuracy. In our study that used senior radiation oncologists' contours as the benchmark, contours of the DLAC by junior radiation oncologists from 11 centers achieved a higher Dice coefficient and smaller MDTA than those achieved by the MC, which indicated that the assistance of deep learning improved the accuracy of their contours.

Regarding the automatic segmentation of a target specifically for lung cancer, we noted that all of the published studies assessed the contour accuracy of the gross tumor on the basis of MR or PET, with the reported Dice coefficient ranging from 0.71 to 0.93 (15–18). To the best of our knowledge, the present study is the first to evaluate the performance of a DLAC of the CTV in the context of planning the CT. By using a DLAC on the planning CT, our study revealed a favorable Dice coefficient of 0.75 for the CTV, which showed an improvement compared to the Dice coefficient of the MC. This moderate Dice coefficient may be explained by the following reasons. First, PORT-CTV generally encompasses the high-risk nodal regions and bronchial stump, which cannot be simply identified by discriminating tissue density as it was for GTV. Second, CT-based postoperative changes may augment anatomical diversity, such as a blurred soft tissue boundary, shifted target location resulting from different lobectomies, and a wide variety of individual patient's lung volume. Third, the PORT-CTV definition is more complex than an anatomical issue. With the development of more sensitive diagnostic techniques and modern conformal radiation techniques, there is a trend toward a smaller PORT volume, which only includes high-risk draining lymph node regions plus the bronchial stump (34). As a result, PORT-CTV may vary with the position of the primary tumor and the examined nodal regions because of differences in nodal drainage, which would inevitably affect the agreement of the training data and consequently decrease the performance of the deep-learning model. The accuracy of the DLAC is expected to increase with an enlarged training dataset and multimodality images.

Inter-observer variability in the CTV contour has been considered to be the decisive source of uncertainty in radiotherapy treatment planning (35, 36). Such variability has become increasingly critical in the context of precision radiotherapy because other sources of error are minimized through advances in radiation technique (37). In the setting of the postoperative CTV contour for NSCLC, significant inter-clinician variations have been observed even among experts (34). The auto-contoured target volumes have been found to be more consistent than the manually contoured volumes in many contexts (13, 38–40). In the present study, with the assistance of DLC, we gained significantly improved consistency of contours across observers. The inter-observer variability among the junior radiation oncologists was decreased significantly in terms of both volumetric and spatial metrics, with the variability reduction rate ranging from 30 to 35%.

Another important issue in target delineation is time consumption. With a prophylactic intent of radiation, the contouring time for PORT depends mainly on three factors: visualization of the boundary of the target, anatomical knowledge of the lymph node regions, and comprehension of the regions with a high-risk of failure. When comparing the DLAC directly with the MC, it should be equally easy to visually distinguish the high-contrast edges, whereas the latter two factors may be better contemplated through the DLAC. The most evident advantage of the DLAC is that it directly offers a possible delineation solution, and the physician only needs to edit the contour rather than to manually contour by slice. In our study, employment of DLAC achieved a 35% reduction of the time consumption for the CTV contour, leading to a considerable improvement in working efficiency.

We acknowledge several limitations in our study. First, the patients enrolled for model establishment were treated over a certain time span, during which there might be certain changes throughout the process of the treatment planning. For instance, several anatomical protocols for the mediastinal lymph node region have been proposed within the recent decade, such as the IASLC lymph node map, Japan consensus, and Michigan atlas (41–43), which would have caused uncertainty in the CTV design to a certain extent within these years and consequently affected the model performance. Second, the planning CT images were collected retrospectively. The CT data could have been better with a thinner slice thickness and the inclusion of breathing information provided by 4D CT. Additionally, all sets of the junior radiation oncologists' contours were completed within 1 month, so a memory bias from the previous contour might have been introduced and could have further affected the delineation of CTV for patients with the latter sequence.

## Conclusions

The DLAC is a promising strategy offering superior accuracy, consistency, and time saving for PORT-CTV delineation, which leads to higher quality and efficiency of radiotherapy for NSCLC after complete resection. These results indicate that deep learning-based automatic segmentation is promising for assisting CTV delineation, particularly for junior physicians from low patient volume institutions.

## Data Availability Statement

The datasets generated for this study are available on request to the corresponding author.

## Ethics Statement

The studies involving human participants were reviewed and approved by Ethics Committee of National Cancer Center/Cancer Hospital, Chinese Academy of Medical Sciences and Peking Union Medical College. Written informed consent for participation was not required for this study in accordance with the national legislation and the institutional requirements.

## Author Contributions

NB, JW, ZZ, and JD contributed conception and design of the study. NB, JW, and ZZ independently contoured the CTVs to generate the ground truth. NB and JW organized the database and performed the statistical analysis and wrote the first draft of the manuscript. NB, JW, TZ, and XC wrote sections of the manuscript. XC, WX, JM, and JD established the deep learning segmentation model. KX, LWu, and QF collected the original data of the study patients. LWa, YL, and ZZ treated and selected the study patients. All authors contributed to manuscript revision, read, and approved the submitted version.

### Conflict of Interest

The authors declare that the research was conducted in the absence of any commercial or financial relationships that could be construed as a potential conflict of interest.
