# Supplementary material for: Deep Learning Improved Clinical Target Volume Contouring Quality and Efficiency for Postoperative Radiation Therapy in Non-small Cell Lung Cancer
Source: Front Oncol. 2019 Nov 13;9:1192. doi: 10.3389/fonc.2019.01192 (PMC6863957; doi:10.3389/fonc.2019.01192)
Supplement: Supplementary file 1 [file Table_1.DOCX]

**Supplementary table 1.** Department volume and working years of junior physicians.

| Name Abbreviations | Number of all patients receiving radiotherapy in 2018 | Number of lung cancer patients receiving radiotherapy in 2018 | Working Years |
| --- | --- | --- | --- |
| DDC | 532 | 128 | 2 |
| XW | 200 | 100 | 2 |
| XYZ | 360 | 85 | 2 |
| YBX | 1873 | 468 | 2 |
| CHX | 359 | 96 | 3 |
| SSW | 600 | 380 | 3 |
| JZ | 400 | 80 | 4 |
| NYJ | 130 | 40 | 4 |
| FC | 256 | 28 | 5 |
| QG | 359 | 18 | 5 |
| QZ | 764 | 235 | 5 |
